# Supplementary figures and images for: Pharmacodynamics of Doripenem Alone and in Combination with Relebactam in an In Vitro Hollow-Fiber Dynamic Model: Emergence of Resistance of Carbapenemase-Producing Klebsiella pneumoniae and the Inoculum Effect
Source: Antibiotics (Basel). 2023 Dec 7;12(12):1705. doi: 10.3390/antibiotics12121705 (PMC10741200; doi:10.3390/antibiotics12121705)

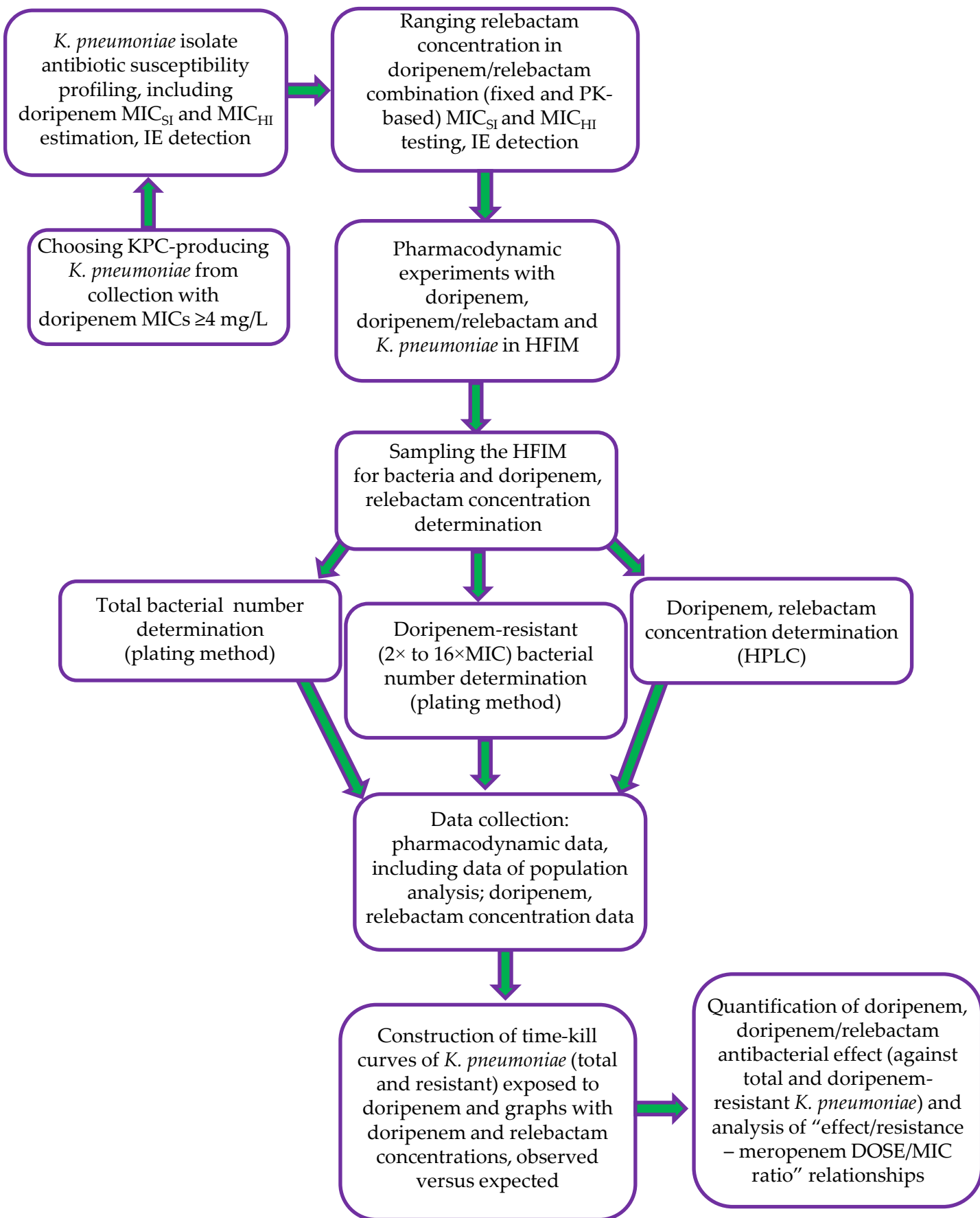

**Figure S1.** The flowchart of the materials and methods section.

Supplement: Supplementary file 1 [file antibiotics-12-01705-s001.zip › antibiotics-2727464-supplementary.pdf]
